# Transcriptional analysis reveals that the intracellular lipid accumulation impairs gene expression profiles involved in insulin response-associated cardiac functionality

**DOI:** 10.21203/rs.3.rs-2688729/v1

**Published:** 2023-04-04

**Authors:** Virginia Actis Dato, María C. Paz, Federico E. Rey, María C. Sánchez, Vicenta Llorente-Cortés, Gustavo A. Chiabrando, Danilo G. Ceschin

**Affiliations:** Universidad Nacional de Córdoba; Universidad Nacional de Córdoba; University of Wisconsin-Madison; Universidad Nacional de Cordoba; Institute of Biomedical Research of Barcelona (IIBB)-Spanish National Research Council (CSIC); Instituto Universitario de Ciencias Biomédicas de Córdoba (IUCBC), Centro de Investigación en Medicina Traslacional Severo R. Amuchástegui (CIMETSA), G.V. al Instituto de Investigación Médica Mercedes y Martín Ferreyra (INIMEC-CONICET-UNC), X5016KEJ – Córdoba; Instituto Universitario de Ciencias Biomédicas de Córdoba (IUCBC), Centro de Investigación en Medicina Traslacional Severo R. Amuchástegui (CIMETSA), G.V. al Instituto de Investigación Médica Mercedes y Martín Ferreyra (INIMEC-CONICET-UNC), X5016KEJ – Córdoba

**Keywords:** LRP1, aggregated low-density lipoprotein, transcriptome analysis, cardiovascular disease

## Abstract

Cardiovascular disease (CVD) is a multisystemic and multicellular pathology that is generally associated with high levels of atherogenic lipoproteins in circulation. These lipoproteins tend to be retained and modified, for example, aggregated low-density lipoprotein (aggLDL), in the extracellular matrix of different tissues, such as the vascular wall and heart. The uptake of aggLDL generates a significant increase in cholesteryl ester (CE) in these tissues. We previously found that the accumulation of CE generates alterations in the insulin response in the heart. Although the insulin response is mainly associated with the uptake and metabolism of glucose, other studies have shown that insulin would fulfill functions in this tissue, such as regulating the calcium cycle and cardiac contractility. Here, we found that aggLDL induced-lipid accumulation altered the gene expression profile involved in processes essential for cardiac functionality, including insulin response and glucose uptake (*lnsr, lns1, Pik3ip1, Slc2a4 gene expression*), calcium cycle (*Cacna1s and Gjc2* gene expression) and calcium-dependent cardiac contractility (*Myh3*), and cholesterol efflux *(Abca1)*, in HL-1 cardiomyocytes. These observations were recapitulated using an in vivo model of hypercholesterolemic ApoE-KO mice. Altogether, these results may explain the deleterious effect of lipid accumulation in the myocardium, with important implications for lipid-overloaded associated CVD.

## Introduction

Cardiovascular disease (CVD) is a multisystem and multicellular pathology frequently associated with increased levels of aggregation-prone small LDL particles (aggLDL) [1–4]. We previously demonstrated that the uptake of aggLDL, through its interaction with the LDL receptor-related protein-1 (LRP1), promotes the accumulation of intracellular cholesteryl ester (CE) and the impairment of the insulin response. This affects the activation of the insulin receptor (IR)/Pl3K/Akt intracellular signalling pathway and the activity of glucose transporter type 4 (GLUT4) in cardiomyocytes [5]. Moreover, in vivo studies have shown that LRP1-mediated CE-enriched lipoprotein uptake results in CE accumulation and decreased insulin response in myocardium of high-fat-diet-fed rabbits (HFD) [6]. Our findings are consistent with extensive evidence linking myocardial steatosis with alterations in insulin signalling, decreased metabolic flexibility, and diastolic dysfunction [7–10]. Insulin response is primarily associated with the uptake and metabolism of glucose [11], which is significance because the heart is a highly metabolic tissue and glucose accounts for 30% of the energy source available for its functions [12]. However, previous studies have also shown that insulin can play a role in other functions in the heart, such as the regulation of calcium cycle and cardiac contractility [13, 14]. Decreased GLUT4 expression and IR activity have been linked to contractile dysfunction and cardiac hypertrophy in mice hearts [13, 14]. Additionally, insulin resistance reduced expression and activity of connexins, leading to alterations in muscle contraction and relaxation cycles [15]. Furthermore, insulin secretion is also associated with increased levels of Cacna1s, a calcium voltage-gated channel involved in calcium influx [16]. In particular, sarcoplasmic reticulum Ca (2+) ATPase (SERCA2) activity, which is crucial for cardiac function, and the conduction of cardiac signals through connexin-40 have been reported to be altered in HL-1 cardiomyocyte cell line exposed to LDL-cholesterol [17] and in hearts of hypercholesterolemic rabbits [18]. However, the effect of lipid accumulation on insulin response, calcium cycle and cardiac function at the transcriptomic level is not yet fully understood. In this study, we conducted a transcriptome analysis of bulk RNA sequencing in samples of HL-1 cardiomyocytes exposed to aggLDL. Our results showed that lipid-overloaded lipoproteins led to lipid accumulation and impaired gene expression related to insulin-induced intracellular signalling, glucose uptake, contractile function, and cholesterol efflux in HL-1 cardiomyocytes. Similar results were obtained in the hearts of hypercholesterolemic ApoE-KO mice. Our findings offer new insights into the negative effects of lipid accumulation on the myocardium at the transcriptomic level, with important implications for lipid-overloaded associated CVD.

## Materials And Methods

### HL-1 cardiomyocyte culture

The HL-1 cardiomyocytes-derived cell line [19, 20] was cultured in Claycomb medium (Sigma-Aldrich) supplemented with 10% fetal bovine serum (FBS), 100 units/ml penicillin, 100 g/ml streptomycin, and L-glutamine 2 mM (lnvitrogen, Buenos Aires, Argentina) on plastic dishes coated with 12.5 g/ml fibronectin and 0.02% gelatine (Sigma-Aldrich), under a 5% CO_2_ atmosphere at 37°C [5]. The cells underwent fasting in culture medium without FBS for 12 h and then incubated with 100 μg/ml aggLDL for 8 h. To compare, HL-1 cardiomyocytes were also treated with insulin 100 nM for 2 h (Sigma-Aldrich) or Claycomb medium vehicle (control) for different times.

### LDL isolation and aggregation

The LDL isolation and aggregation procedures were carried out as previously described [5, 21]. In brief, LDL (density ranging from 1.019 to 1.063 g/ml) was isolated from plasma pools of normocholesterolemic volunteers through sequential ultracentrifugation using KBr gradients (density from 1.019 to 1.063 g/ml). The protein was quantified using the Pierce kit (#23225, ThermoFisher Scientific (Rockford, IL, USA). Aggregation was induced by vortexing LDL in PBS 1X for 5 min, and then centrifuging at 10.000 rpm for 10 min to precipitate the aggLDL. Finally, aggLDL was resuspended with PBS 1X at a concentration of 100 μg/ml.

### mRNA purification and library preparation

HL-1 cardiomyocytes culture was performed with aggLDL, insulin, and vehicle (control) treatments as mentioned. After incubation, I collected the cells using trypsin, centrifugated, and preserved with RNAlater solution (Thermo Fisher Scientific). Total RNA extraction and library preparation was conducted at the Gene Expression Center of the University of Wisconsin Biotechnology Center (UWBC), with four individual experiments for each condition. The samples were subjected to QIAzol Lysis Reagent (Qiagen, Hilden, Germany), followed by phase separation by centrifugation and ethanol treatment. RNA extraction was performed using the RNAeasy protocol with DNase treatment in the column and elution in nuclease-free water. The purity and integrity of each sample were analysed using a NanoDrop One spectrophotometer (Thermo Fisher Scientific, Waltham, MA, USA) and Agilent 2100 Bioanalzyer (Santa Clara, CA, USA). RNA samples with optimal condition [RNA Integrity Number (RIN) > 8] were prepared ac-cording to the TruSeq^®^ Stranded mRNA Sample Preparation Guide (Rev. E) using the lllumina^®^ TruSeq^®^ Stranded mRNA Sample Preparation Kit (lllumina Inc., San Diego, California, USA). For each library, the preparation process involved isolating mRNA from 1,000 ng of total RNA using poly-T oligo-attached magnetic beads. The poly-A-enriched sample was then subjected to fragmentation by treating it with divalent cations at elevated temperature. The resulting fragments were transformed into double-stranded cDNA through a process that involved Superscript II (lnvitrogen, Carlsbad, California, USA), RNaseH, DNA Pol I, and random primers. The cDNA was purified using AMPure XP beads (Agencourt, Beckman Coulter) following of Kienow DNA polymerase incubation, which added an adenine base to the 3’end of the blunt DNA fragments. The fragments were then ligated to unique dual index (UDI) adapters (IDT for lllumina- TruSeq RNA UD Index catalogue 20022371, IDT for lllumina - Nextera DNA Unique Dual Indexes, Set A and custom synthesized UDls), which have a single thymine base overhang at their 3’end. The adapter-ligated DNA products were then purified with AMPure XP beads and amplified in a link-er-mediated PCR reaction (LM-PCR) for 10 cycles using Phusion TM DNA Polymerase and lllumina’s PE genomic DNA primer set, followed by another purification step using AMPure XP beads. Finally, the quality and quantity of the completed libraries were evaluated using Agilent DNA1000 chip (Agilent Technologies, Inc., Santa Clara, CA, USA) and Qubit^®^ dsDNA HS Assay Kit (lnvitrogen, Carlsbad, CA, USA), respectively. The libraries were standardized to 2 nM and Paired-end 2 × 150 bp sequencing was performed on an lllumina NovaSeq6000 sequencer using standard SBS chemistry. The images obtained were analyzed using the standard lllumina Pipeline, version 1.8.2.

### Transcriptomic Analysis

High-quality reads were obtained from different profiles after removing unknown or low-quality bases and adaptor sequences using Trim_galore v0.6.5 (www.bioinformatics.babraham.ac.uk/projects/trim_galore/). The remaining sequences were then mapped to the mouse reference genome (ENSEMBL: mm10.GRCm38.97) using the Subread software v2.0.1 [22]. The quantification of the mapped profiles was performed using the featureCounts tool v1.6.4 [23]. Normalization and differential expression analysis of genes was carried out using the Bioconductor edgeR package v3.28.0 [24], which uses a generalized linear model quasi-likelihood F-test from empirical Bayes methods to estimate gene-specific biological variation [25]. Poorly expressed genes were filtered out by maintaining worthwhile counts in a minimal number of samples. Normalisation using trimmed mean of M values (TMM) was performed to assess relative changes in expression levels between conditions [25]. Negative binomial generalized linear models were fitted to identify differential expression, and differentially expressed genes (DEG) were selected using a logarithmic fold change of 2 and - 2, P-value < 0.01, and a false discovery rate adjusted P-value < 0.05. PCA analysis was performed in using R and plotted using the ggplot2 package v3.3.6, while volcano plots and heatmaps were generated using the EnhancedVolcano package v1.14.0 and the EnrichedHeatmap package v1.26.0, respectively. Gene Ontology (GO) functional enrichment analysis was performed using the web-server g:profller tool [26] and EnrichmentMap plug-ins v3.3.1, ClusterMaker2, and AutoAnnotate v1 .3.3, all running on Cytoscape v3.9.1, were used to view and understand the datasets provided by g:Profiler [27]. The hypothesis-driven pathways were collected using the WikiPathway plugin in Cytoscape, and gene expression values were mapped to the genes in the different pathways.

### Epifluorescence microscopy

HL-1 cardiomyocytes that were cultured on cover glass were washed with PBS 1X, fixed with 4% paraformaldehyde (PFA) for 15 min. Then, either cell or cryosections of mice hearts were incubated with 1 % bovine serum albumin (BSA)- 0.4% Triton, along with boron-dipyrromethene (Bodipy; 3,8 μM) for 20 min. After that, they were incubated with Hoechst 33,258 dye (1/3000) for 10 min. Finally, the samples were mounted using Mowiol 4–88 reagent (Calbiochem Merck KGaA, Darmstadt, Germany). Fluorescent im-ages were obtained using a Leica DMl8 biological microscope (Leica, Germany). 20 photos of cells and 9 photos of cryosections were taken for each condition with 63x magnification. The total fluorescence of the whole cell area (HL-1 cells) or tissue photo area (cryosections) was quantified with lmageJ Fiji software (National Institutes of Health, Bethesda, MD, United States).

### Real Time-PCR

HL-1 cardiomyocytes or heart samples were treated with the TRlzol^®^ reagent (lnvitrogen, Buenos Aires, Argentina) for RNA extraction. One microgram of total RNA was reverse transcribed in a volume of 20 μL [5]. To quantify the transcripts of *lnsr, Ins1, Pik3ip1, Slc2a4, Cacna1s, Gjc2, Myh3, Abca1* and *Gapdh*, the PCR primers listed below were used. The results were normalized to the Gapdh transcripts’ qRT-PCR products. The transcripts were quantified using qRT-PCR (ABI 7500 Sequence Detection System, Applied Biosystems, California, USA) using Sequence Detection software v1.4. The amplification conditions consisted of a 10 min warm up at 95°C, followed by 40 cycles at 95°C for 15 s and 60°C for 1 min. The specificity of the analysis was confirmed through fusion curve analysis and electrophoresis on 2% agarose gel with fluorescence detection using SYBR^®^ Safe DNA (lnvitrogen, Buenos Aires, Argentina). The relative gene expression was calculated using the 2-Ct method, and each sample was analysed three times. No amplification was detected in the PCRs using either water or RNA samples that had not been incubated with reverse transcriptase during cDNA synthesis.

Sequences of mouse primers: *insr* forward 5’-ATGGGCTTCGGGAGAGGAT-3’, *lnsr* reverse 5’-GGATGTCCATACCAGGGCAC-3’; *lns1* forward 5’-TCTACACCCGAGACGAACACT-3’, *lns1* reverse 5’-TGGGCCTTTGCCCGATTATG-3’; *Pi3kip1* forward 5’-CCCAGAGACCACTTCCCAAG-3’, *Pi3kip1* re-verse 5’-TGGTAGGGCGTTAGCAGGA-3’; *Slc2a4* forward 5’-GTGACTGGAACACTGGTCCTA-3’, *Slc2a4* reverse 5’-CCAGCCACGTTGCATTGTAG-3’; *Cacna 1* s forward 5’-TCAGCATCGTGGAATGGAAAC-3’, *Cacna 1* s reverse 5’-GTTCAGAGTGTTGTTGTCATCCT-3’; *Gjc2* forward 5’-TCCACAATCATTCCACCTTCG-3’, *Gjc2* reverse 5’-CAGAAGCGCACATGAGACAG-3’; *Myh3* forward 5’-CCAAAACCTACTGCTTTGTGGT-3’, *Myh3* reverse 5’-GGGTGGGTTCATGGCATACA-3’; *Abca 1* for-ward 5’-CGTTTCCGGGAAGTGTCCTA-3’, *Abca 1* reverse 5’-GCTAGAGATGACAAGGAGGATGGA-3’; *Gapdh* forward 5’-TACCTGCCAGGGCACAAG-3’, *Gapdh* reverse 5’-G GGTACCACAAAAACCAG GA-3’.

### Mouse model

As animal model, we used male C57BL/6J wild-type mice (Wt) and male Apolipoprotein E-deficient (ApoE-KO) mice on the same background (The Jackson Laboratories, Bar Harbor, ME, United States) [28]. We chose to use male mice to avoid fluctuations in the lipid profiles caused by hormonal cycling. The ApoE-KO mice naturally develop metabolic syndrome, including hypercholesterolemia and elevated fasting insulin levels [28]. The mice were housed at a temperature of 22 ± 1°C and were subjected to a 12-h light/12-h dark cycle with free access to water and food (normal commercial mice chow diet). Mice were sacrificed at 6 months of age (Wt; n = 6 and ApoE-KO; n = 6). The hearts of the mice were first perfused with saline solution (0.9% NaCl/1000 U heparin) and then processed for quantitative Real-Time PCR (qRT-PCR), or cryosections. The experimental procedures were approved by the Institutional Animal Care and Use Committee (CICUAL) of the Facultad de Ciencias Químicas, Universidad Nacional de Cordoba (RD-2022–1602-E-UNC-DEC#FCQ), which follows guidelines in compliance with Directive 2010/63/EU. Every effort was made to minimize the number of animals used in agreement with recommendations in the ARRIVE guidelines.

### Cryosection of heart tissue

The hearts of mice were fixed for 24 h in 4% PFA solution at 4°C, followed by overnight incubation in a solution of 30% sucrose in PBS at 4°C. The samples were then embedded in optimum cutting temperature (OCT, TissueTEK, Sakura) compound, and 7-μm-thick radial sections were obtained using a Thermo Scientific Shandon cryostat 0620E. The heart cryosections were stored at - 20 °C under dry conditions and the used for epifluorescence microscopy.

### Statistical analysis

The statistical analysis was performed using the GraphPad Prism 7.0 software. A p-value of less than 0.05 was considered statistically significant. One-way ANOVA followed by Dunnett’s multiple comparisons post-test or Student t-test was used to analyse the data. The data represent the mean ± standard error of the mean (SEM).

## Results And Discussion

**Figure 1:**
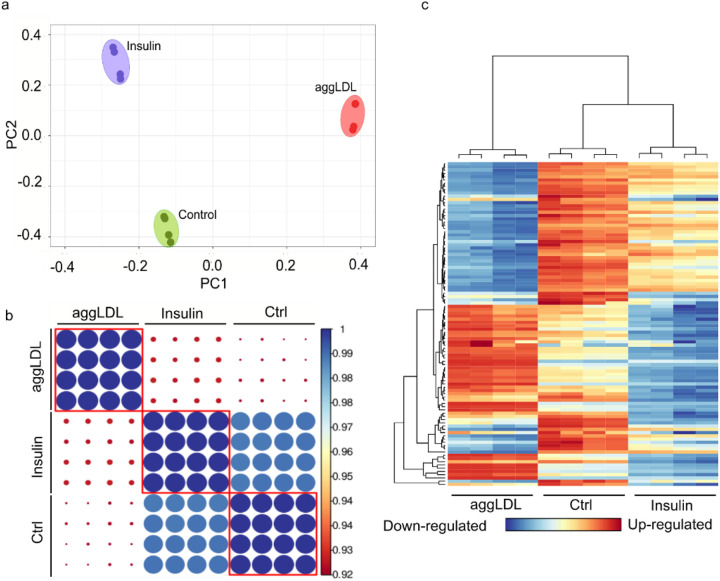
Bulk RNA-Seq was performed on HL-1 cardiomyocytes. The cells were either stimulated with aggLDL 100 μg/ml for 8 h or vehicle (Ctrl) or stimulated with insulin 100 nM for 2h. (a) Principal Component Analysis (PCA), (b) Expression matrix-based Pearson’s correlation analysis, and (c) heatmap representation and unsupervised hierarchical clustering of the top 100 most expressed genes in the transcriptome profiles. Four replicates were performed per condition.

**Figure 2:**
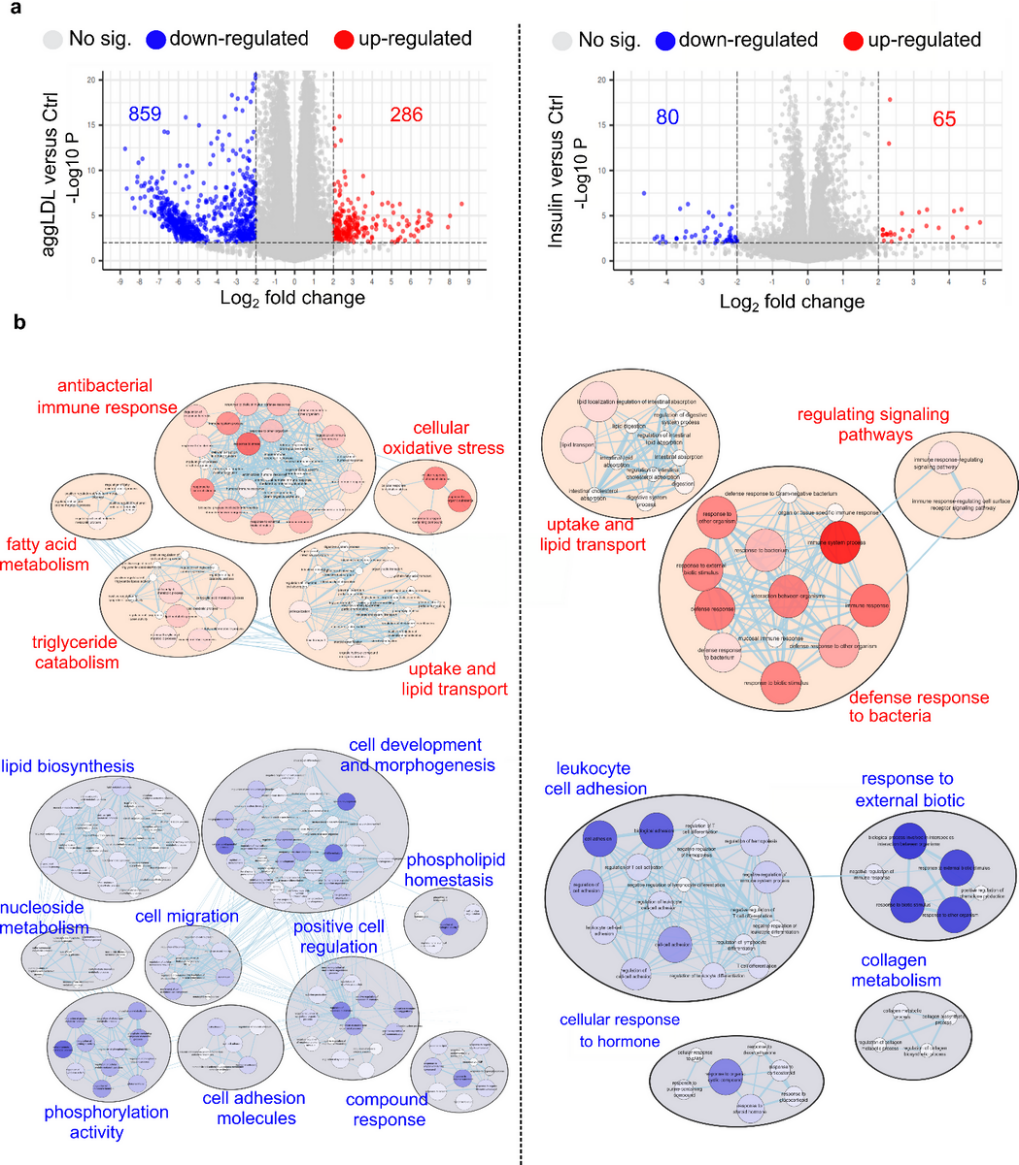
Differentially expressed genes (DEG) analysis between HL-1 treated with aggLDL or insulin compared to vehicle (control) conditions. Left panel corresponds to aggLDL compared to control and right panel correspond to insulin compared to control. (a) Volcano plot shows DEGs with a llogFCl≥2; p-value<0.01; and FDR<0.05. The number of up- and downregulated genes is shown in the graph. (b) Gene Ontology (GO) (Biological Process) enrichment analysis. Red nodes indicate upregulated processes and blue ones down-regulated. The size and intensity of the colours of the nodes are proportional to the number of genes enriching this biological process. The lines connecting the nodes represent the biological processes related to each other.

Insulin plays a critical role in heart development and function, particularly in glucose metabolism [11, 12], as well as cardiac function and contractibility [13–15, 29–32]. In previous studies, we found that CE accumulation impairs insulin action by decreasing insulin intracellular pathway activation and glucose uptake in HL-1 cardiomyocytes and in rabbit myocardium [5, 6]. This impairment could be similar to what occurs in macrophages that uptake oxidized LDL via CD36 and scavenger receptor class A (SR-A), leading to foam cell formation and altered cholesterol homeostasis [33–35]. Additionally, aggLDL uptake promotes CE accumulation in lysosomes by inhibiting hydrolysis in macrophages [36]. Therefore, we investigated the impact of lipid accumulation on insulin response, the calcium cycle, and cardiac function at the transcriptomic level. To evaluate changes in gene expression due to lipid accumulation, we performed bulk RNA-sequencing analysis on HL-1 cardiomyocytes treated with aggLDL (100 μg/ml) for 8 h or vehicle (control). Additionally, to compare the gene expression profile modified by aggLDL to that regulated by insulin, transcriptome analysis was performed on HL-1 cardiomyocytes treated with insulin (100 nM) for 2 h or vehicle (control). We validated the transcriptome profiles through visual inspection of the data, including PCA analysis, Pearson correlation and unsupervised hierarchical clustering with heatmap representation (Fig. 1). Our analysis confirmed methodological repeatability and segregations, with similarity of biological replicates and no outlier samples within each cluster, and the robust segregation between aggLDL, insulin and control conditions (Fig. 1a). The complete expression matrix-based Pearson’s correlation analysis (Fig. 1b) supports these conclusions by showing a positive correlation between all datasets, typically exceeding R = 0.98 among samples belonging to the same cluster. We also performed unsupervised hierarchical clustering on the top100 most expressed genes within the transcriptome profiles, shown by the dendrogram and heatmap (Fig. 1c). The generation of gene unsupervised clusters suggests that genes may be regulated similarly or serve biological functions. The insulin clustering pattern was similar to the control condition (likely due to the short stimulus time), while the aggLDL condition pattern was completely different, as visually apparent in the heatmap. Interestingly, our RNA-Seq dataset showed a completely opposing expression pattern of genes between insulin and aggLDL conditions. Treatment with aggLDL resulted in the upregulation of 286 genes and downregulation of 859 genes relative to the control (Fig. 2a-left panel), while insulin resulted in the upregulation of 65 genes and downregulation of 85 genes relative to control (Fig. 2a-right panel).

The results of the gene ontology analysis comparing aggLDL to control are displayed in the left panel of Fig. 2b. We found upregulated genes belonging to processes such as ‘antibacterial innate immune response’ (related to lipoprotein-induced training immunity in macrophages, monocytes and endothelial cells by oxidized LDL [33, 34], and ‘activation of innate and humeral immune response’, as seen in other modified LDLs [37, 38]). We also observed upregulation of genes involved in ‘cellular oxidative stress’ (increasing gene expression related to reactive oxygen species production, similar to that generated by oxidized and acetylated LDL in macrophages [39]), ‘fatty acid metabolism’, ‘lipid uptake transport’ (related to lipid uptake, transport, and cholesterol esteriflcation for storage in agreement with evidence found in macrophage related to aggLDL effect on CE accumulation in lysosomes by inhibition of its hydrolysis [40], and plasma lipoproteins particle remodelling), and ‘triglyceride catabolism’ (increasing gene expression of lipoprotein and triglyceride lipase activity). We also found downregulated processes mainly involved in ‘cell development and morphogenesis’ (related to muscle cell differentiation and proliferation and tissue development), ‘lipid biosynthesis’ (decreasing gene expression related to cholesterol and fatty acid biosynthesis, and cholesterol efflux), ‘phosphorylation activity’ (related to insulin and Wnt signalling pathways, and regulation of phosphate metabolic process), ‘positive cell regulation’ (including processes such as cell homeostasis, proliferation, apoptosis, muscle differentiation, and calcium cycle, similar to that described for human vascular smooth muscle cells (hVSMC) exposed to oxLDL [41]), ‘cell migration’ (decreasing gene expression involved in cell motility and locomotion), ‘cell adhesion molecules’ (related to expression of cell adhesion molecules), ‘phospholipid homeostasis’ (involving process such as cell junction organization and regulation of cell structure size), and ‘nucleoside metabolic process’. It is notable that the number of biological processes affected by downregulated genes was higher compared to upregulated genes. This may be because the number of downregulated genes was three times higher than the number of upregulated genes (as seen in Fig. 2a-left panel). Furthermore, we confirmed the impairment in insulin signalling gene expression in agreement with our previous evidence in HL-1 cardiomyocytes [5]. We also evaluated our hypothesis-driven by mapping the gene expression levels into mouse WikiPathways [42]. We selected insulin signalling, (WP65, https://www.wikipathways.org/index.php/Pathway:WP65), calcium regulations in cardiac cells (WP553, https://www.wikipathways.org/index.php/Pathway:WP553), striated muscle contraction (WP216, https://www.wikipathways.org/index.php/Pathway:WP216), and cholesterol metabolism (WP4346, https://www.wikipathways.org/index.php/Pathway:WP4346). The logFC mapped to the different WikiPathways can be found in Supplementary Table 1. Despite the majority of downregulated genes in certain pathways, such as insulin signalling, having a logFC value greater than - 2, we were able to identify several downregulated genes within the same pathway. Thus, the cumulative effects of those genes could explain the impairment of the pathway.

In the comparison between insulin and control, we found significant upregulation of genes mainly involved in ‘absorption and transport of lipids’ (increasing gene expression for lipogenic enzymes, fatty acid transport, and intestinal lipid absorption and lipid efflux), ‘regulating signalling pathways’ (insulin and Wnt pathways gene expression are also promoted) and ‘defence response to the bacteria’ (increasing gene expression related to innate immune response to bacteria, in agreement with evidence about insulin-enhanced immune response and trained immunity in macrophage [40]). On the other hand, downregulated genes were involved in processes such as ‘leukocyte cell adhesion’ (decreasing gene expression involved in cell adhesion, T cell differentiation and activation), ‘response to external biotic’ (related to the biological process involved in interspecies interaction between the organism and chemokine production), ‘cellular response to hormone’ (decreasing gene expression involved in cellular response to cAMP, cellular response to glucocorticoid, corticosteroids and steroid hormones), and ‘collagen metabolism’ (by decreasing the expression of metalloproteinase and genes related to collagen biosynthetic pro-cess) (Fig. 2b-right panel).

We also evaluated the combined effect of incubating HL-1 cells with 100 μg/ml of aggLDL for 8 h followed by 100 nM insulin for 2 h. Compared to untreated cells (control), this combined treatment resulted in an upregulation of several processes including ‘lipid biosynthesis’, ‘leukocyte adhesion’, ‘innate immune response’, ‘growth signalling pathway’, ‘lipid esteriflcation’, ‘cholesterol absorption’, and ‘negative regulation of NF-κB signalling’, which could be related to alteration in apoptosis and cell survival. Among the downregulated processes were ‘processing of fatty acids’, ‘calcium homeostasis and cardiac contractibility’, ‘Wnt and insulin signalling of the pathway’, and ‘processing of extracellular matrix or cell motility’ (Supplementary Fig. 1 and Supplementary Table 1). All these up- and downregulated processes were similar to the aggLDL-induced condition, which it may be due to the short duration of the insulin stimulus being insufficient to reverse the effect of lipid accumulation in these cells. Further studies are needed to determine whether insulin can counteract the detrimental of lipid accumulation with longer stimuli.

**Figure 3:**
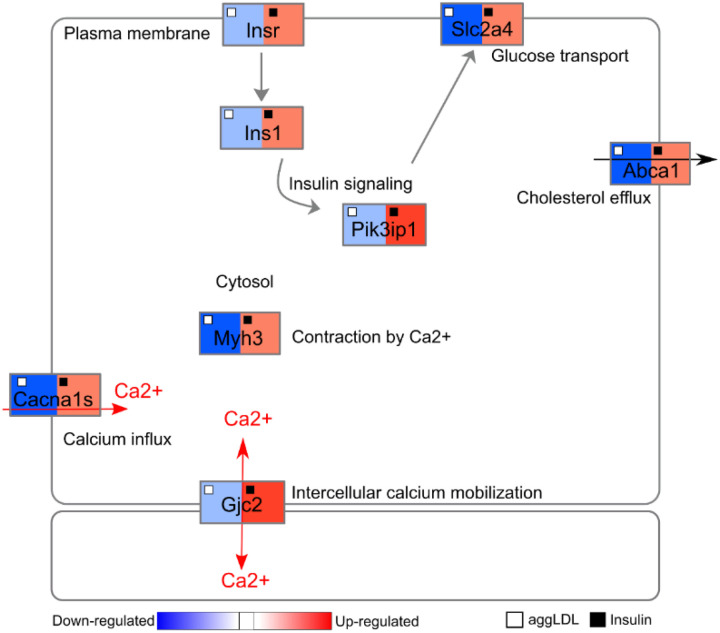
Network diagram of the main intracellular processes affected. Genes involved in different intracellular processes are shown as rectangles in red (upregulated) or blue (downregulated) according to their expression from RNA-Seq profiles under different conditions, insulin 100 nM for 2 h or aggregated LDL 100 μg/ml for 8 h. Conditions are displayed at the top of each rectangle as black (insulin) or white (aggLDL) squares. The network was manually adjusted to show the main genes obtained in the unsupervised GO enrichment and WikiPathways analysis.

Based on the unsupervised GO enrichment and WikiPathways analysis, a network diagram with dysregulated genes of biological processes of interest and logFC values of aggLDL or insulin treatments compared to control is shown in Fig. 3. The focused was on processes related to insulin signalling and glucose uptake (*Insr, Ins1*, *Pik3ip1*, *Slc2a4* gene expression), calcium cycle (*Cacna1s* and *Gjc2* gene expression) and calcium-dependent cardiac contractility (*Myh3*), and cholesterol efflux (*Abca1*), which are well-known processes in the heart.

**Figure 4:**
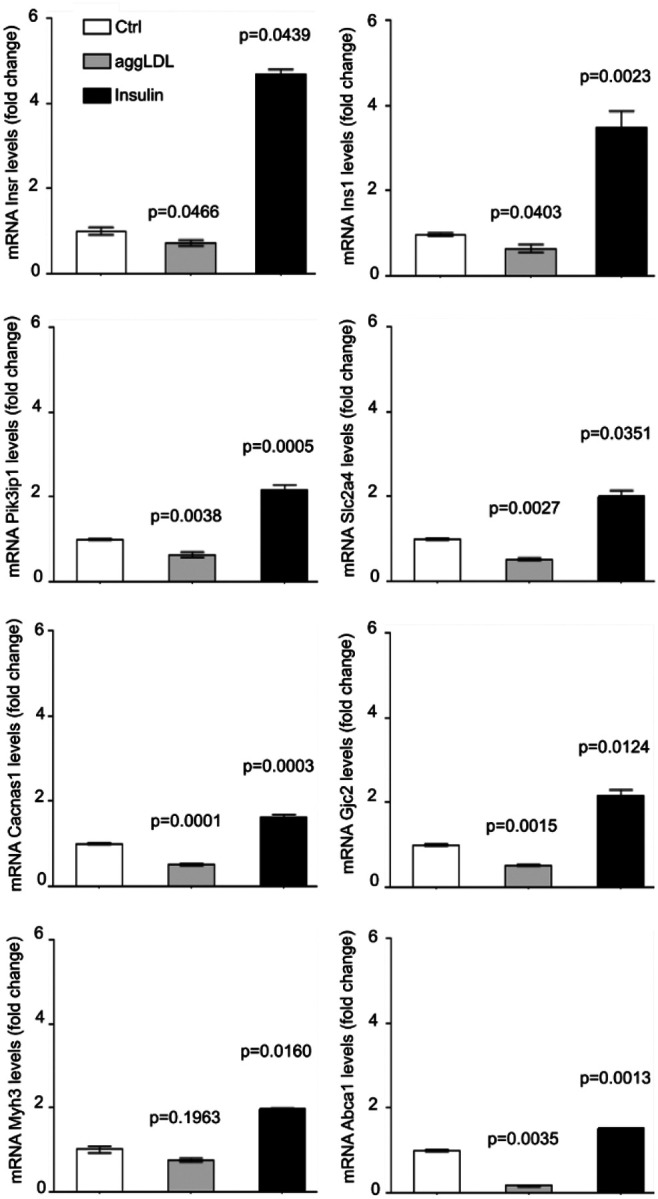
Quantitative RT-qPCR assay to evaluate *lnsr, Ins1, Pik3ip1, Slc2a4, Cacnals, Gjc2, Myh3and* Abca1 mRNA expression in HL-1 cardiomyocytes treated with 100 μg/ml aggLDL for 8 h or insulin 100 nM for 2 h. Bar graph showing the mean ± SEM of mRNA levels, and represented as fold change as compared to the mean of control condition.* p<0.05 are considered significant. The RT-qPCR products of Gapdh transcripts were used as a housekeeping gene loading control.

We found that aggLDL downregulated the expression of these genes, while insulin upregulated them. To validate the diagram in Fig. 3, we performed qPCR validations on fresh samples of HL-1 cardiomyocytes treated with aggLDL (100 μg/ml for 8 h) or insulin (100nM for 2h), same conditions as the transcriptome was done. Our analysis revealed that mRNA expression of the insulin signalling pathway *lnsr*, *lns1*; and *Pik3ip1* (encoding the insulin receptor (IR), insulin receptor substrate-1 (INS1) and phospho-inositide-3- kinase interacting protein 1 (PIK3IP1), respectively), glucose uptake *Slc2a4* (encoding for glucose transporter type 4 (GLUT4)), calcium cycle *Cacna1s* and Gjc2(encoding for calcium voltage-gated channel subunit alpha1 S (CACNA1S) and connexin-47, respectively), calcium-dependent cardiac contractility *Myh3* (encoding miosina-3), as well as cholesterol efflux Abca1 (encoding the ABCA1 transporter) showed a differential expression with a significant reduction by aggLDL and increased expression induced by insulin (Fig. 4). This is in agreement with our previous data that showed that aggLDL effectively increases intracellular lipid accumulation in HL-1 cardiomyocytes (Supplementary Fig. 2) [6]. These results align with previous evidence suggesting that insulin promotes gene expression involved in its own signalling pathway and GLUT4 [29–31], as well as calcium cycling and contractibility [32]. In the present study, the downregulatory effect of aggLDL on these same genes in cardiomyocytes suggests that the intracellular CE accumulation has a deleterious effect on these insulin-regulated processes.

**Figure 5:**
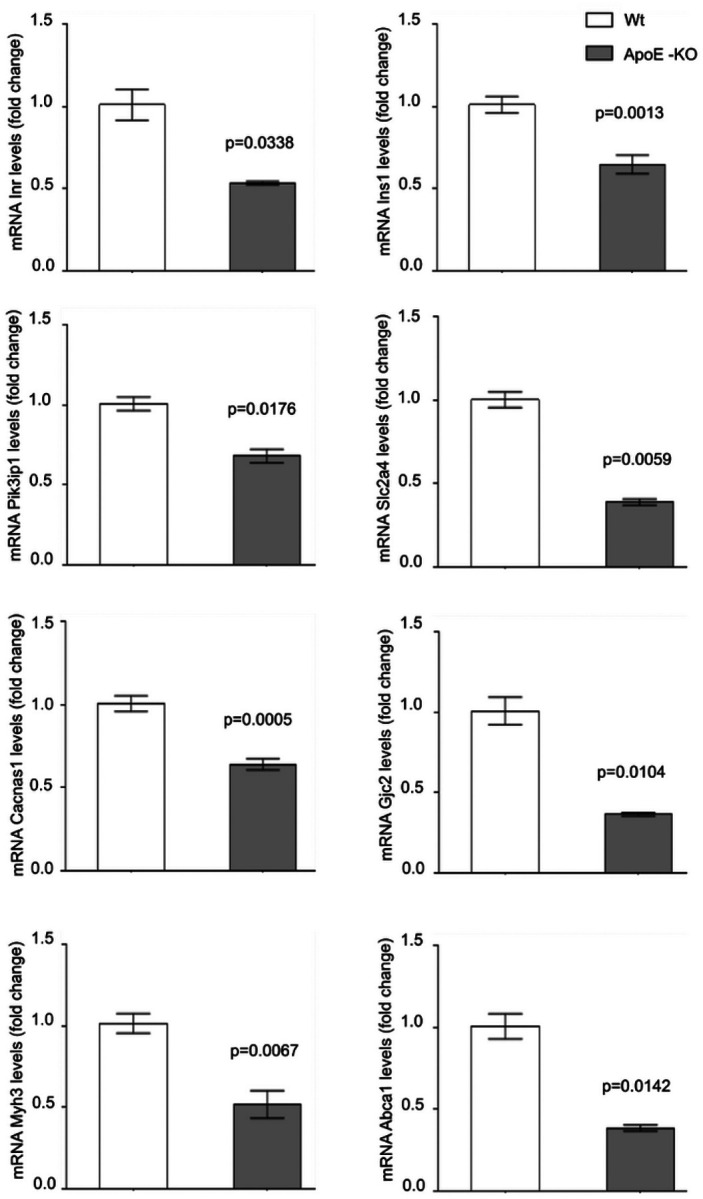
Quantitative RT-qPCR assay to evaluate *lnsr, lns1, Pik3ip1, Slc2a4, Cacna1s*, *Gjc2*, Myh3 and Abca1 mRNA expression in the heart of ApoE-KO or wild-type (Wt) mice of 6 months of age. Bar graph showing the mean ± SEM of mRNA levels, and represented as fold change as compared to the mean of Wt group. * p<0.05 are considered significant. The RT-qPCR products of Gapdh transcripts were used as a housekeeping gene.

Finally, in previous studies we found that ApoE-KO mice had higher levels of circulating cholesterol, triglycerides, LDL-cholesterol, and HDL-cholesterol, and increased fasting plasma insulin levels [28]. The increase in fasting insulin was detected at 6 months of age, while the alteration in the lipid profile (high levels of total cholesterol, HDL and LDL cholesterol) was found at 4 months of age and lasted until 6 months of age [28]. In the present study, we found that ApoE-KO mice (6 months of age) had increased lipid accumulation in the myocardium detected by BODIPV-stained lipid droplet (Supplementary Fig. 3). This suggests that alterations in lipid metabolism precede and, therefore, could be the cause of impaired insulin response. Using qPCR, we characterized the gene expression levels associated with insulin signalling, glucose uptake, calcium cycle, calcium-dependent cardiac contractibility, and lipid efflux in hearts of ApoE-KO mice. Compared to wild-type (Wt) mice, we found that the hearts of ApoE-KO mice had decreased expression of *lnsr*, *lns1*, and *Pik3ip1*(insulin signalling pathway), *Slc2a4* (glucose uptake), Cacna1s and *Gjc2* (calcium cycle), *Myh3* (calcium-dependent cardiac contractility), and *Abca1* (cholesterol efflux) genes (Fig. 5). The decreased gene expression of *Abca1* in hearts of ApoE-KO mice is consistent with the increased lipid accumulation in the myocardium (Supplementary Fig. 3).

## Conclusion

Our studies have revealed that aggLDL leads to intracellular CE accumulation and promotes significant changes in the transcriptome of HL-1 cardiomyocytes. We found that the downregulated genes were around three times more than upregulated genes, impacting several biological processes. In particular, aggLDL decreased expression level of genes involved in insulin signalling and glucose uptake, calcium cycle, myocardial contraction, and cholesterol efflux, including *lnsr*, *Ins1*, *Pi3kip1*, *Sc/2a4*, *Cacna1s, Gjc2, Myh3*, and *Abca1* in HL-1 cardiomyocytes. This expression profile was also validated *in vivo* in hearts from ApoE-KO mice, which exhibited evident accumulation of neutral lipids in myocardium. These findings support the harmful effects of lipid accumulation in the heart and sheds new light on how aggLDL modulates gene expression associated with impaired insulin response, promoting cardiovascular disease. Thus, this highlights the crucial role of insulin response and lipid metabolism in cardiovascular health and emphasizes the need for further research to better understand the mechanisms underlying these dysregulations.

## Limitations

Despite the limitations of our study, we believe that our findings provide valuable insights into the molecular mechanisms underlying insulin response and glucose uptake in cardiomyocytes treated with aggLDL. We acknowledge that further research is needed to validate our results, including protein expression analysis, as well as assessments of metabolism and functional aspects. However, these aspects are beyond the scope of the current study and are currently under development. Additional treatment times could be evaluated to examine different gene clusters in relation to the relevant biological processes. Furthermore, co-staining of lipid and vesicle markers could be performed to better understand the cellular localization of the lipid and their metabolism. These analyses would require a significant amount of time and could potentially extend the manuscript beyond its intended scope. Nevertheless, we believe that our findings could have significant implications for the development of new therapeutic strategies for cardiovascular disease.

## Data Availability

The raw data generated in this study can be accessed through the NCBI Short Read Archive (SRA) using the accession ID PRJNA896115 and the direct link https://www.ncbi.nlm.nih.gov/bioproject/PRJNA896115. The scripts and the data files used for analysis can be found in the following repository: https://github.com/daniloceschin/aggLDL-accum-HL1.
